# Personality and quality-of-life improvement after apomorphine infusion in Parkinson’s disease

**DOI:** 10.1093/braincomms/fcae181

**Published:** 2024-05-24

**Authors:** Mathilde Boussac, Estelle Harroch, Christel Barthelemy, Fabienne Ory-Magne, Clémence Leung, Margherita Fabbri, Christophe Arbus, Christine Brefel-Courbon

**Affiliations:** Toulouse NeuroImaging Center, University of Toulouse, Inserm, UPS, 31024 Toulouse, France; Department of Clinical Pharmacology and Neurosciences, Parkinson Expert Center, Clinical Investigation Center, University Hospital of Toulouse, NeuroToul COEN (Center of Excellence in Neurodegeneration), 31024 Toulouse, NS-PARK/FCRIN Network, France; Department of Clinical Pharmacology and Neurosciences, Parkinson Expert Center, Clinical Investigation Center, University Hospital of Toulouse, NeuroToul COEN (Center of Excellence in Neurodegeneration), 31024 Toulouse, NS-PARK/FCRIN Network, France; Toulouse NeuroImaging Center, University of Toulouse, Inserm, UPS, 31024 Toulouse, France; Department of Clinical Pharmacology and Neurosciences, Parkinson Expert Center, Clinical Investigation Center, University Hospital of Toulouse, NeuroToul COEN (Center of Excellence in Neurodegeneration), 31024 Toulouse, NS-PARK/FCRIN Network, France; Department of Clinical Pharmacology and Neurosciences, Parkinson Expert Center, Clinical Investigation Center, University Hospital of Toulouse, NeuroToul COEN (Center of Excellence in Neurodegeneration), 31024 Toulouse, NS-PARK/FCRIN Network, France; Toulouse NeuroImaging Center, University of Toulouse, Inserm, UPS, 31024 Toulouse, France; Department of Clinical Pharmacology and Neurosciences, Parkinson Expert Center, Clinical Investigation Center, University Hospital of Toulouse, NeuroToul COEN (Center of Excellence in Neurodegeneration), 31024 Toulouse, NS-PARK/FCRIN Network, France; Toulouse NeuroImaging Center, University of Toulouse, Inserm, UPS, 31024 Toulouse, France; Psychiatry Department of the University Hospital of Toulouse, CHU Purpan, 31024 Toulouse, France; Toulouse NeuroImaging Center, University of Toulouse, Inserm, UPS, 31024 Toulouse, France; Department of Clinical Pharmacology and Neurosciences, Parkinson Expert Center, Clinical Investigation Center, University Hospital of Toulouse, NeuroToul COEN (Center of Excellence in Neurodegeneration), 31024 Toulouse, NS-PARK/FCRIN Network, France

**Keywords:** Temperament and Character Inventory, precision medicine, therapeutics

## Abstract

People with Parkinson’s disease with motor fluctuations can be treated by continuous subcutaneous apomorphine infusion (CSAI) to reduce their symptoms. Nonetheless, factors are lacking to predict patients’ quality-of-life amelioration after CSAI. This pilot study aimed to evaluate associations between personality dimensions and quality-of-life improvement after 6 months of CSAI.

Thirty-nine people with Parkinson’s disease awaiting CSAI were included. Linear regression models between ‘Temperament and Character Inventory’ personality dimensions at baseline and percentage of change in Parkinson’s Disease Questionnaire-39 scores after 6 months of CSAI were realized (*n* = 35). The Temperament and Character Inventory was also compared between patients awaiting CSAI and patients awaiting deep brain stimulation of the sub-thalamic nucleus (*n* = 39 from the PREDI-STIM study).

Higher reward dependence scores were associated with a better quality-of-life outcome after 6 months of CSAI, while self-directedness scores were associated with a better quality of life before CSAI (as opposed to harm avoidance, reward dependence and self-transcendence scores associated with a worse quality of life). Moreover, people with Parkinson’s disease awaiting deep brain stimulation of the sub-thalamic nucleus had similar Temperament and Character Inventory dimensions compared to patients awaiting CSAI.

People with Parkinson’s disease with higher reward dependence scores at baseline had the best quality-of-life improvement after 6 months of CSAI. This finding could be used to better prepare and accompany people with Parkinson’s disease during CSAI establishment. Moreover, this result could serve as an orientation factor to second-line treatments.

## Teaser

Boussac *et al*. report that the personality of reward dependence is associated with a better improvement in quality of life after 6 months of CSAI in Parkinson’s disease patients. In a personalized medicine approach, patients with high reward dependence could be preferentially oriented towards this apomorphine infusion.

## Graphical Abstract

**Graphical abstract:**
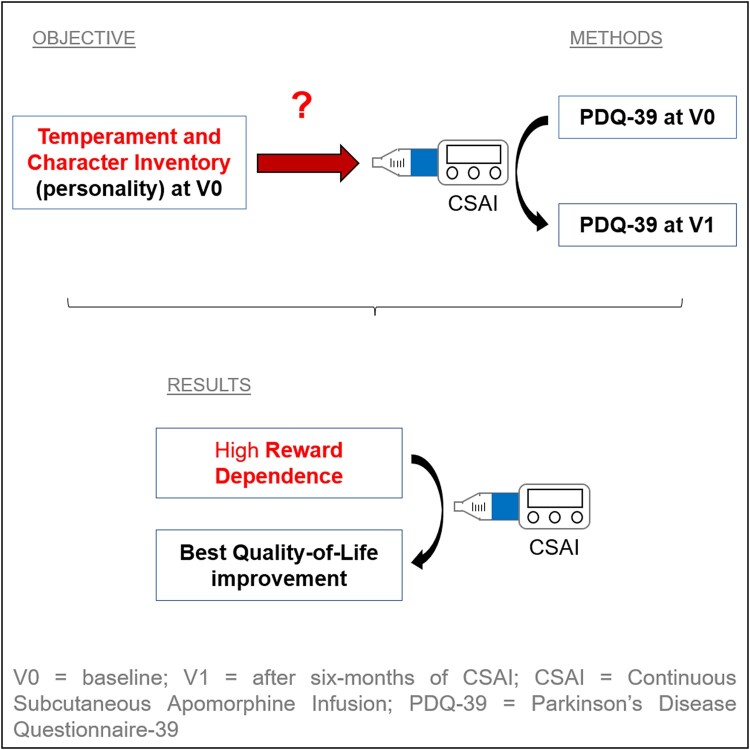

## Introduction

Second-line treatments such as deep brain stimulation of the sub-thalamic nucleus (DBS-STN) or continuous subcutaneous apomorphine infusion (CSAI) can be offered to people with Parkinson’s disease (PwPD) with motor fluctuations.^1^ CSAI significantly reduces OFF time in PwPD compared with placebo, but did not improve quality of life (QoL) in the double-blind, randomized TOLEDO study,^2^ even if some open-label studies have found QoL amelioration following CSAI.^3-7^ Improvements in motor function, overall non-motor burden, sleep, fatigue, mood, apathy and executive functions were also observed after 6 months of CSAI in a recent prospective study.^8^ Similarly, CSAI reduced motor fluctuations and improved QoL without impacting cognition and psychiatric well-being.^7^ Finally, recent reviews confirmed all these results: improvement in OFF-time duration and in various non-motor symptoms (including neuropsychiatric disorders) following CSAI, as well as its safety and efficacy in people with advanced Parkinson’s disease.^9,10^

Personality corresponds to each individual characteristic associated with behaviour, cognition and emotions in order to adapt to the environment,^11^ and it affects the perception of the impact of illnesses on well-being and capabilities.^12^ Hence, personality was shown to be associated with health-related QoL in different studies,^13^ as previously reported in Parkinson’s disease.^14^ In this respect, personality could be an interesting factor for predicting QoL improvement after CSAI, as it was associated with QoL improvement after DBS-STN^15^ using the Temperament and Character Inventory (TCI) (validated in a large cohort of PwPD^16^), which provides a quantitative measure of seven personality dimensions.^17^

In the absence of randomized controlled trials comparing the improvement induced by second-line treatments (DBS-STN versus CSAI),^1^ patients’ choice and clinical judgement remain the main factors in treatment choice.^1^ Since our previous study showed that PwPD with higher novelty seeking (NS) and cooperativeness scores had the best improvement of QoL after DBS-STN,^15^ personality could help to select the best treatment.

This pilot study aimed to determine the associations between personality dimensions and QoL improvement after 6 months of CSAI and as a secondary objective to evaluate associations between personality dimensions and QoL before CSAI establishment. As a complementary objective, using additional data from our previous study, we aim to compare personality dimensions between two cohorts of PwPD awaiting CSAI or DBS-STN.

## Materials and methods

### Patients

In this PSYCHO-PERF study, we included PwPD who started CSAI treatment from 2019 to 2022 at the University Hospital of Toulouse. CSAI introduction was decided in routine care after medical evaluation.

PwPD presenting an atypical parkinsonian syndrome, a DBS device, a significant psychiatric disorder, or cognitive decline [Montreal Cognitive Assessment (MoCA) score < 24] were excluded.

All patients gave their informed consent, and the PSYCHO-PERF study (ClinicalTrials.gov NCT03793491) was approved by the ethics committee (CPP Nord-Ouest IV).

As a complementary objective, supplementary data from the PREDI-STIM cohort (Protocol 2013-A00193–42; ClinicalTrials.gov NCT02360683) of the best-matched PwPD awaiting DBS-STN were extracted and used to compare patients with Parkinson’s disease awaiting CSAI or DBS-STN. The protocol can be found in our previous study.^14^ Matching with PwPD awaiting CSAI was done according to age, disease duration and sex. When a ‘perfect match’ was not available, we prioritized age and sex for matching, followed by only age andonly disease duration. Age and sex were chosen as matching criteria since both factors have been shown to influence TCI personality dimensions scores in different populations such as the normative French one^18^ and different psychiatric populations.^17^  Supplementary Table 1 presents the matched characteristics of all the patients.

### Methods

The primary objective of this pilot study was to evaluate associations between TCI personality dimensions and QoL improvement measured by the change in the PDQ-39 scale after 6 months of CSAI. The secondary objectives were to determine associations between TCI personality dimensions and QoL before starting CSAI treatment and to compare personality dimensions of PwPD awaiting CSAI to those awaiting DBS-STN from the PREDI-STIM study.^14^

Motor and non-motor states [Movement Disorder Society-Unified Parkinson’s Disease Rating Scale (MDS-UPDRS)^19^ in ON condition], depression [Hamilton Depression Rating Scale (HAMD)^20^], anxiety [Hamilton Anxiety Rating Scale (HAMA)^21^], apathy [Lille Apathy Rating Scale (LARS)^22^] and QoL [Parkinson’s Disease Questionnaire-39 (PDQ-39)^23^] were collected at baseline (V0) and 6 months after CSAI (V1). The PDQ-39, a self-questionnaire specific to PD, is divided into eight sub-scales of QoL with a mental component score (‘emotional well-being’, ‘stigma’, ‘social support’, ‘cognitions’, and ‘communication’) and a physical component score [‘mobility’, ‘activities of daily living’ (ADL), and ‘bodily discomfort’], altogether forming a PDQ-39 total score. A higher score indicates a worse QoL.

The TCI was used to assess patients’ personality across seven dimensions^17^ before CSAI (V0) and was not re-assessed after second-line treatment establishment, as in our previous study within the DBS-STN cohort.^15^ This self-questionnaire of 226 items with binary responses (true/false) is divided into four genetically determined temperaments [NS, harm avoidance (HA), reward dependence (RD) and persistence (P)] and three characters, which are developed through growing [self-directedness (SD), cooperativeness (C) and self-transcendence (ST)]. Each dimension score represents a part of an individual personality on a spectrum. The higher the score, the higher the patient presents the associated personality dimension.

The daily total levodopa equivalent dose (LED) was calculated for each patient according to levodopa treatments, dopaminergic agonists, COMT and MAO inhibitors and other antiparkinsonians,^24^ in accordance with the updated systematic review and proposals from Jost *et al*. (2023).^25^

### Statistical analyses

For descriptive analyses, mean, standard deviation and range (min, max) were calculated for quantitative variables, while headcount and percentage were calculated for qualitative variables. Before the analyses, Shapiro tests were done to verify the variables’ normality.

Concerning clinical, behavioural and motor evolution assessment after 6 months of CSAI, we compared the PDQ-39 total score and eight sub-scores, the four parts of the MDS-UPDRS, the HAMD, the HAMA, the LARS and the LED, between V0 and V1 using paired two-sample Mann–Whitney tests or paired two-sample *t*-tests.

Univariate linear regression models were used to evaluate associations between TCI dimensions (explicative variables) and (i) PDQ-39 scores at V0 and (ii) percentages of change in PDQ-39 at V1 (response variables). Due to the small sample size and in order to avoid over-adjustment in the models, Pearson correlations were performed between each TCI dimension and each potentially relevant variable (age, disease duration, LED, MoCA, MDS-UPDRS, LARS, HAMA and HAMD). Only variables with significant correlations (*P*-value < 0.05 and Pearson correlation coefficient ≥ 0.5) were then used as adjustment variables in the models.

Each PDQ-39 sub-score and total score at V0 and the percentages of change of each PDQ-39 sub-score and total score at V1 were separately used as response variables while scores of each TCI dimension were used as explanatory variables, with previous significant correlated variables being used as adjustment. A false discovery rate (FDR) correction was applied for seven comparisons with a calculation of *q*-values (FDR-adjusted *P*-values) for each model.

TCI dimensions and clinical parameters (age, disease duration, LED, PDQ-39, MDS-UPDRS, LARS, HAMA and HAMD) were compared between PwPD with motor fluctuations awaiting CSAI or DBS-STN using two-sample Mann–Whitney tests or two-sample *t*-tests.

All analyses were conducted on R Studio Software Version 2022.02.3, and a threshold of bilateral statistical significance of 0.05 was used. FDR corrections were applied (*q*-values) and interpretations were made carefully according to results strength.

## Results

Forty PwPD with motor fluctuations awaiting CSAI were screened and 39 were included in the PSYCHO-PERF study (1 patient refused the CSAI). At V1, we analyzed 35 patients because of four drop-outs related to CSAI adverse effects [fatigue (*n* = 1), cutaneous intolerance (*n* = 1), hallucinations (*n* = 1) and nausea (*n* = 1)]. Demographic and clinical parameters are presented in Table 1.

**Table 1:** Characteristics of PwPD awaiting CSAI or DBS-STN

	CSAI-PD patients		V0 versus V1—*P*-value [95% CI of the difference]^a^	DBS-STN-PD patients	CSAI versus DBS-STN—*P*-value [95% CI of the difference]^b^
Variables	V0 (*n* = 39)	V1 (*n* = 35)		V0 (*n* = 39)	
**Sex F/M [*N* (%)]**	17 (44%)/22 (56%)	15 (43%)/20 (57%)	/	16 (41%)/23 (59%)	**/**
**Age (years)**	66.4 ± 6.4	**/**	**/**	65.1 ± 5.3	0.25 [−1; 4]
**Disease duration (years)**	10.7 ± 3.7	/	/	10.5 ± 3.5	0.83 [−1.4; 1.8]
**Total LED (mg/day)**	1008.9 ± 401.4	1452.1 ± 449	**8e-7 [−569.5; −287.3]**	1318.7 ± 436.4	**0.002 [−495; −113]**
**Pump LED (mg/day)**	/	716.6 ± 178.9	/	/	**/**
**Oral LED (mg/day)**	1008.9 ± 401.4	735.5 ± 354.2	**8e-6 [186.6; 390.8]**	/	**/**
**MDS-UPDRS-I**	11.8 ± 5	12.5 ± 5.7	0.23 [−3; 0.5]	10.3 ± 5.4	0.26 [−1; 3]
**MDS-UPDRS-II ON**	14.2 ± 5.9	14.6 ± 6	0.54 [−2.2; 1.2]	7.3 ± 6.4	**1e-5 [5; 10]**
**MDS-UPDRS-III ON**	23.7 ± 12.1	16.2 ± 12.9	**0.008 [2.5; 11.5]**	15.9 ± 9.1	**0.004 [2.6; 12.9]**
**MDS-UPDRS-IV**	9.3 ± 2.5	6.4 ± 3.4	**0.002 [1; 3.8]**	9.5 ± 3.5	0.73 [−1.7; 1.2]
**CGI-S**	/	2.3 ± 0.7	/	/	/
**PDQ-39 total**	33.1 ± 12.2	27.4 ± 10.3	**0.005 [1.7; 8.4]**	30.1 ± 12.4	0.29 [−2.6; 8.5]
**Mobility**	38.7 ± 21.3	33.8 ± 21.3	0.18 [−2.1; 10.7]	36.6 ± 20.1	0.66 [−7.3; 11.4]
**ADL**	35.6 ± 18.3	33.2 ± 16.7	0.14 [−1.2; 8.1]	38.5 ± 19.8	0.51 [−11.5; 5.7]
**Emotional well-being**	41.5 ± 20.6	30.8 ± 20	**0.004 [4.2; 16.7]**	29.9 ± 15.7	**0.007 [3.3; 19.8]**
**Stigma**	31.3 ± 19	19.3 ± 17.6	**0.001 [6.3; 18.8]**	32.5 ± 25.5	0.88 [−12.5; 12.5]
**Social support**	12.5 ± 13.6	8.1 ± 13.6	0.22 [−4.2; 16.7]	8.8 ± 15	0.07 [0; 8.3]
**Cognition**	31.9 ± 17.3	28.8 ± 16.3	0.46 [−3.1; 6.7]	26 ± 16.6	0.13 [−1.7; 13.6]
**Communication**	28.6 ± 21.4	24.8 ± 18.7	0.47 [−4.2; 16.7]	25 ± 19.4	0.42 [−8.3; 16.7]
**Bodily discomfort**	44.7 ± 20.9	40.2 ± 22.6	0.23 [−3.2; 12.7]	43.8 ± 20.5	0.62 [−8.3; 16.7]
**LARS**	−27.2 ± 4.3	−27.8 ± 4.1	0.25 [−1; 2.5]	−27 ± 4.6	0.88 [−2.2; 1.9]
**HAMD**	8.8 ± 4.6	6.6 ± 4	**0.006 [0.6; 3.2]**	4.8 ± 4.2	**4e-5 [3; 6]**
**HAMA**	10.2 ± 4.9	8.6 ± 4.8	**0.03 [0; 2.5]**	4.9 ± 5.1	**3e-6 [4; 7]**
**TCI dimensions**					
**Novelty Seeking (NS)**	17.3 ± 5	/	/	15.5 ± 4.4	0.09 [−0.3; 3.9]
**Harm Avoidance (HA)**	18.8 ± 6.4	/	/	17.6 ± 6.1	0.42 [−1.7; 4]
**Reward Dependence (RD)**	15.4 ± 3.2	/	/	14.5 ± 3.9	0.25 [−0.7; 2.5]
**Persistence (P)**	5.4 ± 1.6	/	/	5.5 ± 1.7	0.84 [−1; 1]
**Self-Directedness (SD)**	31.7 ± 6.4	**/**	**/**	34 ± 5.8	0.10 [−5.1; 0.4]
**Cooperativeness (C)**	32.5 ± 5.1	/	/	33.4 ± 4.6	0.18 [−3; 1]
**Self-Transcendence (ST)**	14.2 ± 5.7	/	/	14.9 ± 6.7	0.70 [−4; 2]

Mean ± SD. ^a^Paired two-sample Mann–Whitney tests or *t*-tests between V0 and V1. ^b^Two-sample Mann–Whitney tests or *t*-tests between PwPD awaiting CSAI or DBS-STN; significant *P*-values in bold; PD, Parkinson’s disease; CSAI, continuous subcutaneous apomorphine infusion; DBS-STN, deep brain stimulation of the sub-thalamic nucleus; CI, confidence interval; F/M, female/male; LED, levodopa equivalent dosage; MDS-UPDRS, =Movement Disorder Society-Unified Parkinson’s Disease Rating Scale; ON, medication state; ADL, activities of daily living; LARS, Lille Apathy Rating Scale; HAMD, Hamilton depression scale; HAMA, Hamilton anxiety scale; TCI, Temperament and Character Inventory.

Due to the COVID-19 pandemic and lockdown in France during part of the study (from 17 March to 11 May 2020), five PwPD were evaluated at V1 through phone calls. Nonetheless, this unplanned evaluation did not seem to impact the result of our study since comparisons of data at V1 between patients evaluated in the hospital center and by phone calls did not reveal any significant difference.

At V1, a significant reduction in oral LED, MDS-UPDRS-III-ON, MDS-UPDRS-IV, PDQ-39 total scores, PDQ-39 ‘emotional well-being’ and ‘stigma’ sub-scores, HAMD and HAMA scores were found. Only total LED (CSAI + oral medications) significantly increased (Table 1).

We only found a significant Pearson correlation between P dimension and disease duration at V0 (Supplementary Table 2). Therefore, disease duration was used as an adjustment variable in the linear regression models using P as an explanatory variable.

Table 2 presents the models with significant associations (FDR-corrected or not). At V0, after FDR correction, significant positive associations remained between ST and PDQ-39 total and ‘communication’ scores, between RD and PDQ-39 ‘ADL’ scores and between HA and PDQ-39 ‘emotional well-being’ scores. Conversely, SD and PDQ-39 ‘emotional well-being’ scores were significantly negatively associated (Table 2). At V1, after FDR correction, only RD tended to be negatively associated with the percentage of change in the PDQ-39 total score (Table 2).

**Table 2:** Significant associations between TCI dimensions at V0 and PDQ-39 scores at V0 (*n* = 39) and percentages of change in PDQ-39 at V1 (*n* = 35)

PDQ-39 scale and sub-scales		TCI dimensions	Coefficients	*P*-value	*q*-value	*R*²
**PDQ-39 at V0**	**Total**	**SD**	−0.63	0.04	0.13	0.09
		**ST**	0.98	0.003	**0**.**02**	0.19
	**Mobility**	**ST**	1.35	0.02	0.17	0.11
	**ADL**	**RD**	2.87	0.001	**0**.**009**	0.22
	**Emotional well-being**	**HA**	1.58	0.002	**0**.**007**	0.22
		**SD**	−1.54	0.002	**0**.**007**	0.21
	**Stigma**	**HA**	1.12	0.02	0.06	0.12
		**SD**	−1.11	0.02	0.06	0.12
	**Social support**	**C**	−0.93	0.03	0.20	0.10
	**Cognition**	**SD**	−0.91	0.04	0.08	0.09
		**C**	−1.22	0.02	0.08	0.11
		**ST**	1.26	0.01	0.06	0.15
	**Communication**	**RD**	2.31	0.03	0.11	0.09
		**ST**	1.62	0.006	**0**.**04**	0.16
	**Bodily discomfort**	**C**	1.57	0.02	0.11	0.13
**Changes in PDQ-39 (%) at V1**	**Total**	**RD**	−4.73	0.008	**0**.**05**	0.17
	**ADL**	**P**	−12.71	0.03	0.20	0.09

Significant *q*-values (FDR-corrected *P*-value) in bold; ADL, activities of daily living; SD, self-directedness; ST, self-transcendence; RD, reward dependence; HA, harm avoidance; C, cooperativeness; P, persistence.

No significant difference in the TCI was found between PwPD awaiting DBS-STN and patients awaiting CSAI (Table 1), while significant clinical differences were found between both populations. Patients awaiting CSAI had lower LED, higher MDS-UPDRS II and III, PDQ-39 ‘emotional well-being’, HAMD and HAMA scores at baseline.

## Discussion

Our study suggests that PwPD with higher RD scores at baseline have a better QoL after 6 months of CSAI while SD scores are associated with a better QoL before CSAI (as opposed to HA, RD and ST scores associated with poorer QoL at baseline). Moreover, PwPD awaiting CSAI or DBS-STN had similar personality dimensions.

In accordance with the literature,^5^ after 6 months of CSAI, our study confirmed a significant improvement of motor symptoms (MDS-UPDRS-III), motor complications (MDS-UPDRS-IV) and a significant decrease of daily oral LED.^26^ Global and mental/emotional parts of QoL were also significantly improved after CSAI, as in the literature,^3,4,6,26^ as well as anxio-depressive state, again in line with the literature.^5^

Concerning our main objective, RD was associated with QoL changes after CSAI: higher baseline RD scores were associated with best QoL improvement. Reward dependence corresponds to social and affective dependency with a need for social approval leading to sensible, loving and devoted individuals.^17^ In this model of personality, RD is related to the norepinephrine system,^17^ as shown mainly by genetic studies.^27-29^ Although this model is questionable, there is no direct evidence that RD can be linked to the dopaminergic system, making it difficult to establish a direct link with the role of apomorphine. In addition, apomorphine was also shown to interact with the norepinephrine system.^30,31^ Hence, an interaction between different neurotransmitter systems may certainly be involved in our results. Moreover, this personality dimension has already been associated with a better social part of QoL or a higher perception of social support in different patient populations.^32,33^ Therefore, due to its link with social dependence, PwPD with higher RD before CSAI may have better social adaptation and are so less disrupted by wearing CSAI, allowing them to enjoy a better QoL after CSAI implementation. Conversely, some patients do not appreciate the CSAI because of its inconveniences/constraints^26^ (pump wearing, daily infusion often carried out by a nurse or spouse, etc.), which can lead to a negative feeling of lack/loss of independence and therefore a poor perception of QoL. This dissatisfaction with the CSAI may result in the discontinuation of the pump^34^ for personal reasons,^26^ and poor compliance with the device.^35^ Moreover, a lack of patient support (importance of ‘full-time caregivers’) has been related to CSAI discontinuation in some cases,^36^ which may reveal the potential loss of independence associated with this treatment. As a result, patients who are more socially adapted (higher RD) may be less affected by these disadvantages as they enjoy being cared for.

Furthermore, it is interesting to note that better QoL improvement after CSAI for patients with high RD was found for the overall QoL (PDQ-39 total), rather than for the social component usually found in the literature.^32^

Moreover, despite the worst motor and anxio-depressive state in PwPD awaiting CSAI compared to the ones awaiting DBS-STN, TCI personality dimensions were similar between both cohorts of fluctuating PwPD awaiting second-line treatments. These demographical differences (LED, motor and anxio-depressive state) may be explained by surgical selection criteria.

The main limitation of our study is the small sample size. Thus, to confirm the results of this pilot study, we are currently conducting a multicentric study evaluating the relationships between several bio-psycho-social factors (personality, beliefs about the treatment, ways of coping and social support) and the improvement in QoL after CSAI (ClinicalTrials.gov NCT06080399). Patient-reported outcome measures are also another innovative and important way of conducting research that we will consider in our future studies since it could have a major impact on advanced therapies.^37,38^ Another limitation could be the absence of behavioural variables (such as the anxio-depressive state) as adjustment in our models. However, due to the small sample size, we only used relevant variables correlated with TCI personality dimensions in order to avoid over-fitting in the models. In addition, we are aware that a significant number of analyses were performed to evaluate the associations between the seven personality dimensions and the QoL improvement after CSAI. Nevertheless, the use of the FDR correction for each personality dimension comparisons should validate the reliability of our positive results. We aimed to assess personality dimensions as predictors of improved QoL, and, therefore, they were only assessed prior to the implementation of the intervention. Any personality changes induced by CSAI could be the subject of another study.

Finally, since we found different associations between personality and QoL outcome after CSAI and DBS-STN,^15^ personality dimensions could be used as a predictive factor of QoL improvement to orientate patients towards their best second-line treatment. PwPD with higher RD scores could be preferably oriented towards CSAI, while PwPD with higher NS and cooperativeness scores could be preferably oriented towards DBS-STN.^15^ To implement this guideline in clinical practice, our team is currently validating a decision algorithm based on personality to best guide PwPD towards DBS-STN.

## Supplementary Material

fcae181_Supplementary_Data

## Data Availability

The data supporting the findings of this study are available on request from the corresponding author.
