# Supplementary material for: Personality and quality-of-life improvement after apomorphine infusion in Parkinson’s disease
Source: Brain Commun. 2024 May 24;6(3):fcae181. doi: 10.1093/braincomms/fcae181 (PMC11154139; doi:10.1093/braincomms/fcae181)
Supplement: fcae181_Supplementary_Data [file fcae181_supplementary_data.docx]

**SUPPLEMENTARY DATA**

**Supplementary table 1**. Characteristics (age, disease duration and sex) of all the matched PD-patients awaiting either CSAI or DBS-STN (n=39)

| **ID of PD patients** | | **Age** | | **Disease duration** | | **Sex** | |
| --- | --- | --- | --- | --- | --- | --- | --- |
| **CSAI** | **DBS-STN** | **CSAI** | **DBS-STN** | **CSAI** | **DBS-STN** | **CSAI** | **DBS-STN** |
| 01 | 02-077 | **65** | **65** | 11 | 10 | **Male** | **Male** |
| 02 | 02-031 | 72 | 71 | 16 | 13 | Female | Male |
| 03 | 17-038 | 70 | 69 | **16** | **16** | **Male** | **Male** |
| 04 | 05-001 | **65** | **65** | 6 | 7 | **Male** | **Male** |
| 05 | 02-051 | **68** | **68** | 16 | 12 | **Female** | **Female** |
| 06 | 19-010 | **64** | **64** | **12** | **12** | **Female** | **Female** |
| 07 | 05-032 | **67** | **67** | 11 | 13 | **Male** | **Male** |
| 08 | 01-042 | **59** | **59** | 14 | 9 | **Male** | **Male** |
| 09 | 13-019 | **67** | **67** | **10** | **10** | **Male** | **Male** |
| 10 | 06-045 | **73** | **73** | 11 | 6 | Male | Female |
| 11 | 17-035 | 70 | 69 | 12 | 13 | **Male** | **Male** |
| 12 | 01-029 | **67** | **67** | 7 | 9 | **Female** | **Female** |
| 13 | 13-035 | **57** | **57** | **6** | **6** | **Female** | **Female** |
| 14 | 15-025 | **68** | **68** | **12** | **12** | **Male** | **Male** |
| 15 | 06-066 | **55** | **55** | 9 | 13 | **Female** | **Female** |
| 16 | 05-028 | 77 | 67 | **14** | **14** | **Female** | **Female** |
| 17 | 20-011 | **72** | **72** | 12 | 7 | **Male** | **Male** |
| 18 | 02-026 | **69** | **69** | 8 | 16 | **Male** | **Male** |
| 19 | 01-026 | 74 | 64 | **8** | **8** | **Male** | **Male** |
| 20 | 01-030 | 77 | 66 | **14** | **14** | **Female** | **Female** |
| 21 | 05-019 | **68** | **68** | 13 | 5 | **Female** | **Female** |
| 22 | 05-003 | **60** | **60** | **7** | **7** | **Male** | **Male** |
| 23 | 06-014 | **65** | **65** | 10 | 8 | **Male** | **Male** |
| 24 | 17-011 | **68** | **68** | **7** | **7** | **Male** | **Male** |
| 25 | 13-028 | **50** | **50** | **5** | **5** | **Male** | **Male** |
| 26 | 17-014 | **59** | **59** | **8** | **8** | **Male** | **Male** |
| 27 | 02-027 | **67** | **67** | 18 | 14 | **Female** | **Female** |
| 28 | 01-020 | 79 | 61 | **12** | **12** | **Male** | **Male** |
| 29 | 06-073 | **55** | **55** | 9 | 11 | **Female** | **Female** |
| 30 | 11-014 | **69** | **69** | 15 | 12 | **Female** | **Female** |
| 31 | 06-025 | **70** | **70** | 8 | 11 | **Female** | **Female** |
| 32 | 02-043 | **69** | **69** | **7** | **7** | Female | Male |
| 33 | 02-041 | **69** | **69** | 8 | 13 | **Male** | **Male** |
| 34 | 05-027 | **68** | **68** | 5 | 9 | **Female** | **Female** |
| 35 | 02-042 | **63** | **63** | 9 | 8 | **Male** | **Male** |
| 36 | 06-038 | **71** | **71** | 15 | 17 | **Female** | **Female** |
| 37 | 04-015 | **60** | **60** | **8** | **8** | **Male** | **Male** |
| 38 | 10-022 | **57** | **57** | **8** | **8** | **Male** | **Male** |
| 39 | 17-081 | 68 | 66 | **19** | **19** | **Female** | **Female** |

*Identical matching in bold; PD = Parkinson’s Disease; CSAI = Continuous Subcutaneous Apomorphine Infusion; DBS-STN = Deep Brain Stimulation of the Sub-Thalamic Nucleus.*

*Concerning matching: n=31 PD patients are age-matched; n=7 disease duration-matched; n=36 sex-matched; n=10 matched for age, disease duration and sex; and n=36 matched for at least two characteristics (age and/or disease duration and/or sex).*

**Supplementary table 2**. Correlations between TCI dimensions and variables at V0 (n=39)

| **TCI dimensions** | **Variables (V0)** | **Coefficients** | **p-value** | **CI 95%** |
| --- | --- | --- | --- | --- |
| **Novelty Seeking** | **Age** | 0.03 | 0.86 | -0.29; 0.34 |
|  | **Disease duration** | 0.03 | 0.84 | -0.29; 0.35 |
|  | **LED** | 0.03 | 0.87 | -0.29; 0.34 |
|  | **MoCA** | -0.06 | 0.74 | -0.36; 0.27 |
|  | **MDS-UPDRS-I** | -0.06 | 0.70 | -0.38; 0.26 |
|  | **MDS-UPDRS-II ON** | -0.17 | 0.29 | -0.46; 0.15 |
|  | **MDS-UPDRS-III ON** | -0.23 | 0.16 | -0.52; 0.1 |
|  | **MDS-UPDRS-IV** | -0.08 | 0.62 | -0.39; 0.24 |
|  | **LARS** | -0.14 | 0.39 | -0.44; 0.18 |
|  | **HAMD** | 0.05 | 0.76 | -0.27; 0.36 |
|  | **HAMA** | 0.04 | 0.79 | -0.28; 0.36 |
| **Harm Avoidance** | **Age** | 0.07 | 0.69 | -0.26; 0.37 |
|  | **Disease duration** | 0.13 | 0.42 | -0.19; 0.43 |
|  | **LED** | -0.07 | 0.67 | -0.38; 0.25 |
|  | **MoCA** | 0.20 | 0.22 | -0.12; 0.49 |
|  | **MDS-UPDRS-I** | 0.23 | 0.17 | -0.1; 0.51 |
|  | **MDS-UPDRS-II ON** | 0.06 | 0.73 | -0.26; 0.37 |
|  | **MDS-UPDRS-III ON** | 0.00 | 0.99 | -0.33; 0.32 |
|  | **MDS-UPDRS-IV** | 0.01 | 0.97 | -0.31; 0.33 |
|  | **LARS** | 0.02 | 0.93 | -0.31; 0.33 |
|  | **HAMD** | 0.18 | 0.27 | -0.14; 0.48 |
|  | **HAMA** | 0.36 | 0.03* | 0.04; 0.61 |
| **Reward Dependence** | **Age** | -0.26 | 0.11 | -0.53; 0.06 |
|  | **Disease duration** | -0.10 | 0.56 | -0.4; 0.23 |
|  | **LED** | 0.20 | 0.22 | -0.12; 0.49 |
|  | **MoCA** | 0.17 | 0.29 | -0.15; 0.46 |
|  | **MDS-UPDRS-I** | 0.18 | 0.28 | -0.15; 0.47 |
|  | **MDS-UPDRS-II ON** | 0.25 | 0.12 | -0.07; 0.53 |
|  | **MDS-UPDRS-III ON** | 0.27 | 0.11 | -0.06; 0.55 |
|  | **MDS-UPDRS-IV** | 0.01 | 0.96 | -0.31; 0.33 |
|  | **LARS** | -0.20 | 0.22 | -0.49; 0.13 |
|  | **HAMD** | 0.04 | 0.83 | -0.29; 0.35 |
|  | **HAMA** | 0.19 | 0.26 | -0.14; 0.48 |
| **Persistence** | **Age** | -0.29 | 0.07 | -0.56; 0.03 |
|  | **Disease duration** | **-0.57** | 0.0001* | -0.75; -0.31 |
|  | **LED** | -0.07 | 0.65 | -0.38; 0.25 |
|  | **MoCA** | 0.06 | 0.73 | -0.26; 0.37 |
|  | **MDS-UPDRS-I** | 0.05 | 0.77 | -0.27; 0.36 |
|  | **MDS-UPDRS-II ON** | -0.22 | 0.19 | -0.5; 0.11 |
|  | **MDS-UPDRS-III ON** | -0.19 | 0.26 | -0.48; 0.14 |
|  | **MDS-UPDRS-IV** | -0.01 | 0.95 | -0.33; 0.31 |
|  | **LARS** | -0.33 | 0.04* | -0.59; -0.01 |
|  | **HAMD** | -0.05 | 0.78 | -0.36; 0.28 |
|  | **HAMA** | -0.09 | 0.59 | -0.4; 0.24 |
| **Self-Directedness** | **Age** | -0.23 | 0.16 | -0.51; 0.09 |
|  | **Disease duration** | -0.37 | 0.02* | -0.62; -0.07 |
|  | **LED** | -0.10 | 0.55 | -0.4; 0.22 |
|  | **MoCA** | 0.02 | 0.89 | -0.29; 0.34 |
|  | **MDS-UPDRS-I** | -0.27 | 0.10 | -0.54; 0.06 |
|  | **MDS-UPDRS-II ON** | -0.10 | 0.53 | -0.41; 0.22 |
|  | **MDS-UPDRS-III ON** | 0.06 | 0.73 | -0.27; 0.38 |
|  | **MDS-UPDRS-IV** | 0.08 | 0.63 | -0.25; 0.39 |
|  | **LARS** | -0.35 | 0.03* | -0.6; -0.04 |
|  | **HAMD** | -0.12 | 0.49 | -0.42; 0.21 |
|  | **HAMA** | -0.36 | 0.02* | -0.61; -0.05 |
| **Cooperativeness** | **Age** | -0.26 | 0.10 | -0.54; 0.06 |
|  | **Disease duration** | -0.06 | 0.70 | -0.37; 0.26 |
|  | **LED** | 0.01 | 0.93 | -0.3; 0.33 |
|  | **MoCA** | 0.11 | 0.52 | -0.22; 0.41 |
|  | **MDS-UPDRS-I** | 0.06 | 0.72 | -0.26; 0.37 |
|  | **MDS-UPDRS-II ON** | 0.09 | 0.60 | -0.24; 0.39 |
|  | **MDS-UPDRS-III ON** | -0.01 | 0.97 | -0.33; 0.32 |
|  | **MDS-UPDRS-IV** | 0.06 | 0.73 | -0.27; 0.37 |
|  | **LARS** | -0.23 | 0.17 | -0.51; 0.1 |
|  | **HAMD** | 0.02 | 0.92 | -0.3; 0.33 |
|  | **HAMA** | -0.08 | 0.62 | -0.39; 0.24 |
| **Self-Transcendence** | **Age** | -0.10 | 0.56 | -0.4; 0.23 |
|  | **Disease duration** | 0.14 | 0.40 | -0.18; 0.44 |
|  | **LED** | 0.07 | 0.66 | -0.25; 0.38 |
|  | **MoCA** | -0.03 | 0.84 | -0.34; 0.29 |
|  | **MDS-UPDRS-I** | 0.23 | 0.16 | -0.1; 0.51 |
|  | **MDS-UPDRS-II ON** | 0.28 | 0.08 | -0.04; 0.55 |
|  | **MDS-UPDRS-III ON** | 0.09 | 0.60 | -0.24; 0.4 |
|  | **MDS-UPDRS-IV** | 0.00 | 0.98 | -0.32; 0.32 |
|  | **LARS** | -0.11 | 0.51 | -0.41; 0.22 |
|  | **HAMD** | 0.36 | 0.03* | 0.05; 0.61 |
|  | **HAMA** | 0.40 | 0.01* | 0.09; 0.64 |

*LED=Levodopa Equivalent Dosage; MoCA=Montreal Cognitive Assessment; MDS-UPDRS=Movement Disorder Society-Unified Parkinson’s Disease Rating Scale; ON = medication state; LARS=Lille Apathy Rating Scale; HAMD=Hamilton Depression scale; HAMA=Hamilton Anxiety scale; CI 95%= Confidence Interval of difference at level of 95%; *p-value<0.05; in bold, absolute coefficient superior to 0.5*
